# Supplementary material for: Pharmacological Properties of Jaeumgeonbi-Tang on Redox System and Stress-Related Hormones in Chronic Subjective Dizziness: A Randomized, Double-Blind, Parallel-Group, Placebo-Controlled Trial
Source: Pharmaceuticals (Basel). 2022 Nov 9;15(11):1375. doi: 10.3390/ph15111375 (PMC9696700; doi:10.3390/ph15111375)
Supplement: Supplementary file 1 [file pharmaceuticals-15-01375-s001.zip › pharmaceuticals-1974349-supplementary.pdf]

Supplementary Materials

Table S1. Biochemistry test results of subjects after 4 weeks of JGT administrations.

Variables	Treatment	JGT	Placebo	Reference range
Total protein (g/dL)	Before (all)	7.31 ± 0.49	7.38 ± 0.39	6.5 ~ 8.5
	2-30s	7.24 ± 0.55	7.54 ± 0.39	6.5 ~ 8.4
	40s	7.25 ± 0.37	7.05 ± 0.26	6.7 ~ 7.8
	5-60s	7.37 ± 0.49	7.36 ± 0.33	7.0 ~ 8.5
	After (all)	7.53 ± 0.41	7.52 ± 0.37	7.0 ~ 8.4
	2-30s	7.60 ± 0.42	7.52 ± 0.36	7.0 ~ 8.3
	40s	7.58 ± 0.47	7.40 ± 0.34	7.0 ~ 8.4
	5-60s	7.47 ± 0.32	7.56 ± 0.35	7.1 ~ 8.0
Albumin (g/dL)	Before (all)	4.40 ± 0.29	4.50 ± 0.29	3.8 ~ 5.0
	2-30s	4.38 ± 0.31	4.73 ± 0.21	4.0 ~ 5.0
	40s	4.32 ± 0.18	4.10 ± 0.21	3.8 ~ 4.5
	5-60s	4.46 ± 0.29	4.44 ± 0.16	4.0 ~ 5.0
	After (all)	4.49 ± 0.26	4.48 ± 0.26	4.0 ~ 5.1
	2-30s	4.55 ± 0.33	4.61 ± 0.25	4.0 ~ 5.1
	40s	4.45 ± 0.24	4.20 ± 0.22	3.9 ~ 4.9
	5-60s	4.47 ± 0.18	4.46 ± 0.18	4.2 ~ 4.7
A/G ratio	Before (all)	1.54 ± 0.18	1.58 ± 0.21	1.1 ~ 1.9
	2-30s	1.54 ± 0.14	1.70 ± 0.15	1.3 ~ 1.9
	40s	1.50 ± 0.22	1.45 ± 0.30	1.1 ~ 1.9
	5-60s	1.55 ± 0.15	1.52 ± 0.13	1.3 ~ 1.8
	After (all)	1.49 ± 0.14	1.49 ± 0.24	1.0 ~ 1.9
	2-30s	1.47 ± 0.15	1.61 ± 0.19	1.2 ~ 1.9
	40s	1.43 ± 0.09	1.38 ± 0.30	1.0 ~ 1.8
	5-60s	1.51 ± 0.13	1.42 ± 0.18	1.0 ~ 1.7
AST (IU/L)	Before (all)	22.17 ± 6.67	20.56 ± 5.12	13 ~ 38
	2-30s	18.42 ± 4.50	18.30 ± 2.45	13 ~ 28
	40s	18.83 ± 2.97	18.50 ± 3.64	13 ~ 23
	5-60s	26.36 ± 6.51	23.36 ± 5.73	15 ~ 38
	After (all)	23.50 ± 6.14	21.24 ± 4.10	15 ~ 38
	2-30s	18.57 ± 1.40	18.6 ± 2.73	15 ~ 23
	40s	22.00 ± 4.83	19.75 ± 3.30	16 ~ 29
	5-60s	27.45 ± 5.71	24.18 ± 3.27	17 ~ 38
ALT (IU/L)	Before (all)	16.67 ± 8.91	16.08 ± 5.56	6 ~ 28
	2-30s	12.14 ± 2.47	14.5 ± 5.52	6 ~ 26
	40s	12.17 ± 2.11	15.25 ± 4.66	9 ~ 22
	5-60s	22.00 ± 10.34	17.82 ± 5.13	10 ~ 28
	After (all)	18.54 ± 9.85	16.92 ± 4.19	9 ~ 38
	2-30s	13.00 ± 2.39	14.60 ± 3.61	10 ~ 21
	40s	16.33 ± 5.06	18.00 ± 2.24	11 ~ 26
	5-60s	23.27 ± 11.85	18.63 ± 4.05	12 ~ 38
ALP (IU/L)	Before (all)	60.13 ± 18.61	57.76 ± 14.49	26 ~ 107
	2-30s	55.57 ± 10.08	52.90 ± 8.62	43 ~ 73
	40s	49.66 ± 18.61	49.00 ± 5.61	26 ~ 87
	5-60s	68.73 ± 17.99	65.27 ± 16.48	37 ~ 107
	After (all)	62.46 ± 18.43	57.60 ± 15.56	30 ~ 105
	2-30s	57.90 ± 6.06	51.30 ± 6.39	43 ~ 64
	40s	55.33 ± 23.74	49.00 ± 5.61	30 ~ 105
	5-60s	19.91 ± 14.77	22.36 ± 19.13	33 ~ 103
γ-GTP (IU/L)	Before (all)	16.54 ± 11.22	19.24 ± 16.24	7 ~ 80
	2-30s	14.29 ± 5.90	18.30 ± 14.14	7 ~ 58

	40s	13.00 ± 1.63	13.00 ± 3.08	10 ~ 17
	5-60s	19.91 ± 14.77	22.36 ± 19.13	9 ~ 80
	After (all)	18.87 ± 11.38	19.68 ± 14.92	8 ~ 74
	2-30s	16.14 ± 6.79	18.80 ± 13.01	8 ~ 54
	40s	15.00 ± 6.81	14.75 ± 5.12	10 ~ 30
	5-60s	22.73 ± 17.73	22.27 ± 17.50	10 ~ 74
Total Cholesterol (mg/dL)	Before (all)	199.29 ± 42.11	185.36 ± 31.86	129 ~ 243
	2-30s	172.86 ± 22.82	171.00 ± 23.96	129 ~ 209
	40s	184.50 ± 19.92	177.00 ± 18.32	146 ~ 204
	5-60s	224.18 ± 44.46	201.45 ± 33.16	139 ~ 243
	After (all)	209.42 ± 49.79	180.76 ± 26.73	133 ~ 259
	2-30s	195.86 ± 41.40	167.30 ± 18.36	140 ~ 259
	40s	191.33 ± 18.34	163.00 ± 16.34	136 ~ 221
	5-60s	227.90 ± 57.20	199.45 ± 22.95	154 ~ 247
Triglyceride (mg/dL)	Before (all)	120.92 ± 134.28	104.76 ± 49.74	33 ~ 256
	2-30s	71.14 ± 21.13	86.00 ± 26.44	41 ~ 121
	40s	85.33 ± 22.51	100.00 ± 30.62	51 ~ 132
	5-60s	172.00 ± 179.62	124.45 ± 60.58	33 ~ 256
	After (all)	114.17 ± 82.67	107.24 ± 51.48	42 ~ 212
	2-30s	72.14 ± 18.51	83.90 ± 29.48	44 ~ 133
	40s	90.67 ± 16.23	130.25 ± 48.06	68 ~ 198
	5-60s	153.73 ± 104.59	120.09 ± 57.43	42 ~ 212
Glucose (mg/dL)	Before (all)	93.29 ± 10.92	92.00 ± 8.50	74 ~ 136
	2-30s	90.14 ± 5.44	89.50 ± 9.69	74 ~ 107
	40s	91.00 ± 4.40	94.75 ± 6.30	84 ~ 102
	5-60s	96.55 ± 14.14	93.27 ± 6.93	82 ~ 136
	After (all)	95.96 ± 10.45	94.46 ± 9.72	63 ~ 112
	2-30s	91.57 ± 10.38	90.30 ± 11.12	63 ~ 106
	40s	96.67 ± 7.13	96.75 ± 5.93	82 ~ 103
	5-60s	98.36 ± 10.67	97.82 ± 7.21	82 ~ 112
Creatinine (mg/dL)	Before (all)	0.77 ± 0.13	0.81 ± 0.14	0.59 ~ 1.09
	2-30s	0.65 ± 0.08	0.80 ± 0.12	0.59 ~ 1.09
	40s	0.82 ± 0.05	0.79 ± 0.06	0.70 ~ 0.85
	5-60s	0.84 ± 0.11	0.84 ± 0.17	0.70 ~ 1.04
	After (all)	0.81 ± 0.10	0.82 ± 0.13	0.59 ~ 1.10
	2-30s	0.71 ± 0.06	0.83 ± 0.12	0.59 ~ 1.10
	40s	0.82 ± 0.05	0.78 ± 0.06	0.72 ~ 0.87
	5-60s	0.87 ± 0.09	0.85 ± 0.16	0.70 ~ 0.99
BUN (mg/dL)	Before (all)	11.83 ± 3.39	12.30 ± 3.64	5.5 ~ 21.8
	2-30s	10.44 ± 2.01	10.50 ± 3.20	5.5 ~ 14.6
	40s	11.70 ± 3.13	13.00 ± 2.00	7.3 ~ 16.9
	5-60s	12.78 ± 3.45	13.67 ± 3.64	7.6 ~ 21.8
	After (all)	11.66 ± 2.53	12.63 ± 3.84	6.0 ~ 22.5
	2-30s	11.56 ± 2.27	10.85 ± 3.64	6.0 ~ 18.6
	40s	9.95 ± 1.67	11.42 ± 1.96	7.7 ~ 14.7
	5-60s	12.66 ± 2.46	14.69 ± 3.34	9.7 ~ 22.5

Data are expressed as mean ± SD. A/G, albumin to globulin ratio; AST, aspartate aminotransferase; ALT, alanine aminotransferase; ALP, alkaline phosphatase; γ-GTP, gamma-glutamyl transpeptidase; BUN, blood urea nitrogen.

Table S2. Hematological test results of subjects after 4 weeks of JGT administrations.

Variables	Treatment	JGT	Placebo	Reference range
WBC (102/μL)	Before	60.33 ± 16.18	58.04 ± 14.07	40 ~ 103
	After	56.58 ± 12.11	58.40 ± 15.54	31~ 95
Segment (%)	Before	55.40 ± 8.17	55.13 ± 7.05	33.5 ~ 66.9
	After	52.72 ± 8.12	55.93 ± 9.16	33.4 ~ 71.8
Monocyte (%)	Before	4.66 ± 2.04	4.84 ± 2.37	1.4 ~ 9.4
	After	5.08 ± 2.28	4.66 ± 2.52	2.0 ~ 8.7
Lymphocyte (%)	Before	41.61 ± 7.20	38.56 ± 5.72	30.9 ~ 54.4
	After	40.56 ± 6.68	38.23 ± 5.43	27.0 ~ 56.6
Hemoglobin (104/μL)	Before	12.94 ± 1.65	12.95 ± 1.37	9.4 ~ 13.9
	After	12.90 ± 1.57	13.0 ± 1.41	9.3 ~ 13.8
RBC	Before	402.6 ± 34.0	404.4 ± 32.4	373 ~ 492
	After	385.2 ± 33.7	399.8 ± 40.6	378 ~ 491
Hematocrit (%)	Before	37.59 ± 7.53	38.83 ± 3.40	29.3 ~ 44.4
	After	37.75 ± 7.51	38.80 ± 3.67	25.7 ~ 44.7
ESR (mm/hr)	Before	11.77 ± 9.61	11.64 ± 8.32	3 ~ 28
	After	11.33 ± 7.80	13.40 ± 9.61	5 ~ 23
Platelets (104/μL)	Before	23.27± 5.46	23.87 ± 5.11	19.3 ~ 31.1
	After	23.99 ± 5.11	24.00 ± 5.71	17.8 ~ 33.6
MCV (fl)	Before	86.95 ± 15.86	89.57 ± 7.30	66.3 ~ 98.0
	After	87.27 ± 14.76	89.52 ± 6.99	66.0 ~ 102.0
MCH (%)	Before	28.93 ± 5.49	29.88 ± 2.76	20.0 ~ 34.7
	After	29.16 ± 5.19	33.32 ± 0.91	20.7 ~ 34.2
RDW (%)	Before	12.72 ± 2.64	12.56 ± 1.33	10.6 ~ 16.6
	After	12.13 ± 2.45	12.72 ± 1.63	10.0 ~ 16.5

Data are expressed as mean ± SD. WBC, white blood cells; RBC, red blood cells; ESR, erythrocyte sedimentation rate; MCV, mean corpuscular volume; MCH, mean corpuscular hemoglobin; RDW, red cell distribution width.

Table S3. Composition of JGT

Herbal name	Scientific name	Part of use	Place of origin	Amount (g)
Gastrodiae Rhizoma	*Gastrodia elata* Bl.	Rhizoma	S. Korea (Muju)	1.2
Adenophorae Radix	*Adenophora triphylla* var. *japonica* Hara	Root	S. Korea (Jinan)	1.2
Hoelen	*Poria cocos* Wolf	Mushroom	S. Korea (Bonghwa)	0.5
Atractylodis Rhizoma	*Atractylodes japonica* Koidz.	Root	S. Korea (Bonghwa)	0.5
Citrus Pericarpium	*Citrus unshiu* Markovich	Peel	S. Korea (Seoguipo)	0.4
Pinelliae Rhizoma	*Pinellia ternata* (Thunb.) Breitenbach	Rhizoma	S. Korea (Seoguipo)	0.4
Paeoniae Radix Alba	*Paeonia lactiflora* Pall.	Root	S. Korea (Andong)	0.4
Rehmanniae Radix	*Rehmannia glutinosa* Liboschitz	Root	S. Korea (Andong)	0.4
Angelicae Gigantis Radix	*Angelica gigas* Nakai	Root	S. Korea (Yeongju)	0.4
Liriope Tuber	*Liriope platyphylla* Wang et Tang	Root	S. Korea (Milyang)	0.4
Cnidii Rhizoma	*Cnidium officinale* Makino	Root	S. Korea (Bonghwa)	0.4
Jujube Fructus	*Zizyphus jujuba* Miller var. *inermis* Rehder	Fruits	S. Korea (Boeun)	0.4
Zingiberis Rhizoma	*Zingiber officinale* Rosc.	Rhizoma	S. Korea (Seosan)	0.4
Schizonepetae Spica	*Schizonepeta tenuifolia* Briquet	Herba	S. Korea (Bonghwa)	0.4
Saposhnikoviae Radix	*Saposhnikovia divaricata* Schischkin	Root	S. Korea (Yeongju)	0.4
Polygalae Radix	*Polygala tenuifolia* Willd.	Root	China (Yunnan)	0.1
Glycyrrhizae Radix	*Glycyrrhiza uralensis* Fisch.	Root	China (Neimenggu)	0.1
Menthae Herba	*Mentha arvensis* Linne var. *piperascens* Malinvaud	Leaf	S. Korea (Jecheon)	0.01
Total				8.0

Table S4. Retention time, linear range, regression equation, correlation coefficient, and contents of seven marker compounds from JGT

Chemical constructions	Compounds	UV (nm)	Retention time (min)	Linear range (μg/mL)	Regression equation (y=ax+b)	Correlation coefficient (r^2)	Contents Mean±SD (μg /mg)
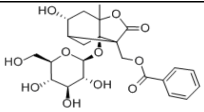	Albiflorin	230	14.3	2.0~150	y = 10.0540 x + 16.169158	0.999	2.0018 ± 0.0402
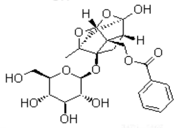	Paeoniflorin	230	15.8	2.0~150	y = 15.0484 x + 11.0123	0.999	92.4079 ± 0.3724
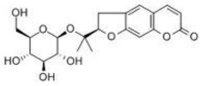	Nodakenin	330	23.7	1.0~75.0	y = 55.2948 x + 70.4863	0.999	0.1465 ± 0.0063
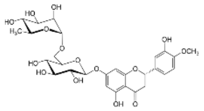	Hesperidin	280	26.9	2.0~150	y = 17.6242 x + 45.6974	0.999	1.1832 ± 0.0981
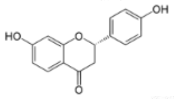	Liquirtigenin	280	33.4	1.0~75.0	y = 37.368 x + 44.9646	0.999	1.0409 ± 0.0544
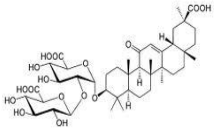	Glycyrrhizinic acid	254	45.3	2.0~150	y = 5.7376 x + 8.2351	0.999	1.1281 ± 0.0739
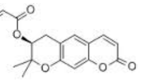	Decursin	330	50.4	1.0~75.0	y = 42.7344 x + 16.0783	1.000	0.0170 ± 0.0009
